# Discussions of the Third Annual Meeting of Central States Association

**Published:** 1866-12

**Authors:** 


### DISCUSSIONS OF THE THIRD ANNUAL MEETING OF CENTRAL STATES ASSOCIATION.

Held at Louisville, July 17, 1866.

### *Wednesday Morning.*

#### TEMPORARY TEETH AND THEIR PRESERVATION.

Dr. MORGAN.—The human being is a unit, and any impediment to this unity tends to impair the perfection of that state more particularly in the case of the child during the progress of the formative processes. The temporary teeth are more important than the permanent, to the proper mastication of food, to aid in building up the general organization for the future. Hence, I say, at this age any loss impairs for life.

How to preserve them is a very different subject. I attribute their early decay to improper nutrition. In removing superficial decay, I find the file a very efficient instrument. Separate freely, exposing the dentine. For filling, if there is danger that the decay will reach the pulp before the tooth is shed, I use amalgam. Have occasionally destroyed pulp in deciduous teeth.

Dr. STONE, feared to use amalgam on account of the danger of absorption by circulation. Inquired of Dr. Morgan if he had ever seen such a case.

Dr. Morgan had seen ptyalism produced by use of amalgam, the mercury being taken up in the circulation. Absorption ceases when vitality is destroyed.

Dr. Stone. Does this premature decay result from natural or local causes? Or is it hereditary? My observation would teach me that the most healthy have the poorest teeth, and *vice versa.* I esteem it of the highest importance, as a preventive of decay, to advise cleanliness. In case of superficial decay, I remove with a file.

Dr. Shadoan. Any method of filling deciduous teeth, so that they are preserved until nature is ready to discard them, is preferable to extracting them. I do not fill pulp cavity with gold, for fear it may, by absorption of the tooth, come into contact with second tooth, and cause more mischief than if let alone. I fill pulp cavity with cotton, that it may yield to pressure of second tooth as it advances. As a preventive there must be something to neutralize the acid of the mouth. The saliva is alkaline, and the mucus is acid. Has noticed that children who sleep with open mouths have an accumulation of dry tartar around the necks of the teeth, which acts chemically to destroy them. It is the accumulation of dried mucus.

Dr. Morgan. In reply to Dr. Stone, would say the case is not a good one; not a fair illustration. Thinks he will find the subjects of a scrofulous diathesis. Such subjects scarletina will sweep, like the besom of destruction, they are the ones first attacked. The old theory of Fox and Bell, as to the sack being a protection to crown of tooth, I consider erroneous; also, that the pressure produces sufficient stimulant to effect absorption of deciduous teeth.

Dr. Goddard. Owing to the liability of the arch to contract if deciduous teeth are prematurely removed, I only extract in extreme cases. Use the file. As an instance of the injury resulting from this practice, I will relate the case of a gentleman whose teeth were very irregular, but prizing highly a well arranged set, was determined that his children should have beautiful teeth, for the accomplishment of which he, as soon as the deciduous set were fairly erupted, commenced to loosen them, and before the children were seven years of age, he had removed them all. When these children had arrived at maturity, one of them had the molars and two front teeth, the other only the molars.

Dr. STONE. In case of sack being formed, if not reducible, I will always extract. Sometimes, the circulation being active, the secretion of the sack may be carried off.

Dr. MORGAN thought that in the cases mentioned by Dr. Goddard the germs were destroyed.

Dr. McCLELLAN. The temporary teeth are equally as important, and as essential to good health as the permanent; for children should as thoroughly masticate their food as the adult. By growth and expansion of the maxillary bones there are frequently spaces formed for the lodgement of food, which, if not removed, produces decay, hence would give great force to the necessity of cleanliness. I use the file, forming v shaved separations. For filling approximal cavities, when decay has gone too far, to be removed with a file, I use the artificial dentine. This will not answer for crown cavities, as it will not stand attrition. If I desire to destroy the pulp, I apply very minute quantities of arsenic. After removing the nerve, I fill fangs with cotton saturated with collodion, the crowns with artificial dentine. Carbolic acid is a good substitute for creosote. When an abscess has formed, after the age of six years, if it is the first temporary molar, I extract. If the second, treat it by injections of diluted iodine. This will generally obliterate the sack. I will endorse Dr. Morgan's statement as to the non-absorption of healthy fangs, unless the second tooth is advancing.

### INDICATIONS FOR THE EXTRACTION OF TEETH.

Dr. McCULLUM gave four requirements:

*First*—Ascertain if tooth indicated, is the one diseased.

*Second*—If the organ is one that can be dispensed with.

*Third*—If the recuperative powers are sufficiently active to restore the diseased organ to health.

*Fourth*—Whether patient is willing to pay, and to follow advice.

Dr. SHADOAN will extract under any of these conditions; where an abscess is incurable; where the presence of the tooth is injurious; when the crown is lost, and the fang is a cause of irritation; when the tooth exostosed; when the dens sapientia is on the angle of the jaw, causing inflammation and prevents proper occlusion; but will never extract for toothache alone—there must be other attendant conditions.

Dr. BAXTER. I have an indication not yet referred to by any speaker. A patient entered my office while suffering from neuralgia of face, and requested me to extract a certain tooth which he supposed to be the offender. From the appearance of the tooth, I positively refused. He went away. Next day he came again, and I again declined extracting it. The third day he returned, this time almost crazy with pain, and angry at me. With many threatening gestures he came towards me, leaving me no alternative but to extract the tooth or receive a thrashing. This I call an active indication for extraction.

Dr. BAXTER presented a case of malformation of the anterior upper teeth, caused he supposed by premature extraction. The right central and lateral were united by an osseous growth. The patient was of scorbutic habits, and of nervo-lymphatic temperament.

Dr. WILSON extracts all teeth where the fangs are exposed; also the first bicuspid in case of crowded teeth. Is opposed to filling childrens teeth, as under the necessary pressure, injury to the process is frequently the result. Had seen teeth forced further into the sockets. Adjourned.

*Afternoon Session.*

SUBJECT RESUMED.

Dr. STONE would always extract when abscess had formed and inflammation is as great as to injure the adjacent teeth, or those of second denture about to erupt.

In case of crowded teeth, it requires close judgment to decide which tooth should be removed, but he always would without hesitation, extract teeth commonly denominated tusks. I do not fully appreciate the merits of the method adopted by many, of enlarging the arch by pressure, or bringing teeth into the arch by various mechanical appliances. This is, perhaps, to be attributable to not having met with success in my few attempts.

Dr. Driggs. I would differ with Dr. Stone, being more conservative. The alveolar arch is, oftentimes, too contracted to favor a good development. In many cases I extract the six year old molars to give room for crowded teeth. For the correcting of irregularities, I frequently resort to mechanical appliances, to restore teeth to their natural position. It seems as though, accepting nature as our guide, we will not remove teeth to make room for others, except in case of decay; for, by observation we will see that in a perfect set they stand as closely to each other as possible.

Dr. Mason. When the cuspids are out of line, I prefer to remove the bicuspids, as the former are too intimately connected with the expression of the face, and their removal causes a falling away of that portion directly over them. Another reason: if the cuspid has erupted above the arch, the removal of a bicuspid gives room, so that it will eventually fall to its proper position.

Dr. McClelland.—Leaves the irregular teeth, if admissible until near the age of puberty, thereby preserving the arch. When circumstances compel the extraction of any tooth, I prefer the second bicuspid, for reason advanced by Dr. Driggs. He gave a case of the incisor tooth, of a boy 12 years of age, broken by a fall into three pieces, nearly longitudinally. To aid in supporting application to the nerve, he let the pieces remain after which the loose portions and the nerve were removed. Treated fang, the foramen of which was yet quite large, with tannin. Saw patient in a few days, when there was a slight indication of pus. Cleansed and filled as before, and in a few weeks filled the fang and fragment of crown with gold, the object being to preserve the space for the reception of an artificial tooth later in life. More than a year has elapsed and the fang has given no trouble.

Dr. MORGAN. Is always governed by circumstances of case. Never extracts canines unless compelled. Which tooth shall be extracted is much governed by the manner of occlusion with lower teeth, as there are cases where the upper teeth cannot charge position for this reason. In such a case would take out the second bicuspid.

GOLD AS A MATERIAL FOR FILLING.

Dr. STONE.—From long practice, I can best manipulate soft foil, in strips or tape. Am not satisfied that so good a filling can be made with adhesive as with soft foil. Have used cylinders, but not with success.

Dr. SHADOAN would recommend each operator to choose the method and the kind of foil best adapted to his ability and skill. He used adhesive.

DR. GODDARD. Until recently have never used other than soft foil in ropes, pellets and tape. Within six months have seen some of them put in eighteen years ago, that are still in good condition, preserving the teeth. Recently have experimented with cylinders, formed by rolling the tape on fine steel wire, also use the mallet for fastening and condensing. With my slight experience I am well pleased with the method.

Dr. NAGHEL. Use soft foil.

Dr. MASON. Use soft foil in strips. In approximal cavities of front teeth, sometimes key the filling with adhesive. I object to the adhesive foil, on account of the impossibility to condense, only in the direction of point of plugger, while soft foil will condense laterally. For condensing surface of filling, I use a thin flat instrument with square corners, resembling a long blade hatchet, introduced between teeth and endwise not with point, but with these corners, making a V shaped impression on the filling, and condensing in every direction.

Dr. McClelland. I have tried most of the methods extant with varied success and failure. I now use adhesive foil and crystal gold with mallet. The foil formed into a rope, by means of crimpers, this is done without handling it, then cut into small pieces. I now use soft foil only in filling fangs. As good a filling, and with the same unity cannot be made with soft, as with adhesive or crystal gold. In large cavities I use the crystal, in the smaller the adhesive as it is more easily introduced into a small space. To work this successfully and to insure all parts of the cavity to be filled, requires a nicety of manipulation not necessary with soft foil.

Dr. Morgan. In reply to Dr. McClelland, answered first, that fillings can be made of soft foil, that will wear like enamel, not yielding a particle to attrition. Secondly, that the softer the foil the more readily will it adapt itself to the cavity. Third, a weaker tooth can be filled with soft than with hard or adhesive foil. I also prefer the soft on account of its lateral expansion in condensing. I have tested the various methods of preparing foil, and have chosen the strips. Occasionally use adhesive in building out a point, or the angles of a crown filling.

I do not object to the use of adhesive foil by others, for some succeed, as I have seen many good fillings, but can and do say that the best of fillings can be and are made of soft foil.

I consider the mallet a labor saving instrument to the dentist, and esteem it valuable in filiing the posterior teeth, as they are more easily accessible with it.

Dr. McClelland wished to be understood by the term soft foil as non-adhesive foil.

Dr. Russell uses soft foil in pellets, carry to cavity and fasten with plugging plyers; then with a smooth pointed instrument condense until the base is firm; after which I use the serrated pluggers.

Dr. McClelland exhibited casts of a mouth taken before and after operating. The natural teeth were worn down almost to the gum. Thirteen teeth had been filled, and elongated with crystal gold and the mallet. The lower incisors were lengthened fully three-sixteenths of an inch, and an eye tooth and bicuspid more than a quarter of an inch. As an exhibition of skill it was much admired, though not looked upon as fully practicable.

THIRD SUBJECT RESUMED.

Dr. Filly a recent advocate for adhesive foil, stated that he had weighed it in the balance, and found it wanting.

Dr. Shadoan, in fillng front teeth where enamel is thin, introduces a piece of foil paper between the gold and the enamel, not letting it extend quite to the edge of enamel as it would, by absorption of fluids of the mouth, produce decay. The only object is to conceal the gold, and present a white appearance.

OTHER MATERIAL THAN GOLD.

Dr. Goddard, has used tin, amalgam, and Wood's alloy. Prizes it very highly as a temporary and cheap filling.

There were none present who used any of the temporary, or cheap materials for filling, where gold could be successfully introduced.

Dr. Morgan uses tin and amalgam, more frequently the latter, in grinding surface, and building up of frail molars, also in children's teeth. Has built up teeth which patients wanted extracted, and would not pay for gold, that have lasted for years. We should be cautious in our remarks commending amalgam, in the presence of young operators, as there is great danger of their being misled.

Dr. Stone. Have used Hill's stopping and find it very valuable in frail cavities. Tin foil should be pure, otherwise it will flake.

Dr. Mason. Use Hill's Stopping largely in such cases as require it. In a few cases use tin foil and os-artificial; but am not satisfied that I can do any better than with gold.

Dr. Cobb. Four years ago I first used amalgam, and am now satisfied that I saved some teeth that I could not with gold. Both have their peculiar advantages—the former certainly has its place as a filling. Wood's metal requires care to make a perfect filling. Endorses Dr. Morgan's remarks as to misleading young operators.

Dr. Mason. Is there a better test than time? As a proof that other material will save teeth, cited two cases. First patient now living. Twenty years ago the nerve of a central incisor being dead, the fang was *plugged* with a hickory stick, and the tooth saved. Second case was of a molar fang, *plugged* with same material, when extracted the stick protruded 1 16 of an inch beyond the apex of fang.

I do not recommend cheap fillings on account of the malpractice frequently seen in this kind of operations, and can see no reason to change.

Dr. Storer. Have never put in an amalgam filling. A few years ago when it was much used, I saw very many of them in country towns adjoining where I was located. They were much discolored. This deterred me from its use, particularly in children's teeth, fearing the absorption of the mercury into the system.

Dr. Morgan stated that Dr. Watt was called out on this absorption and replied that the creosote formed an insoluble compound with the albumen, thus preventing the absorption spoken of by Dr. Stone. Dr. T. J. Clark, of N. O. fills fang with *Hickory* and the crown with gold.

Dr. McClelland uses most of the auxiliary fillings where he thinks they are indicated. Has known artificial dentine (a filling made of gutta percha) to save frail teeth when decayed on approximal surfaces, from five to eight years. It is not hard enough to long withstand the attrition of mastication. Oxychloride of Zinc supports the frail walls of a tooth better than any other material. After it hardens, I excavate sufficiently to fill with gold. *Discolored* teeth may be lightened up better in this way than by almost any other method.

Considers Wood's metal preferable to tin, where it can be introduced *perfectly*. An objection to its use is the liability to leave leaks, when the tooth will turn yellow and decay. It should be introduced in a semi-fused state, filling next to the walls of the cavity first. When he can do as well with other material, never uses amalgam.

In answer to Dr. Cobb, said he could not fill *every* tooth with gold, and does not deem it advisable.

AFTERNOON SESSION.—THIRD DAY.

Dr. McCullum, of Augusta, Ky., read an essay on "the Causes of Decay of Teeth." This was prepared in common with several others for the purpose of reading before the High school of Augusta, hoping by this means to elicit an interest in the children in the preservation of their teeth. It was written in an easy comprehensive style, well adapted to accomplish the desired result.

A vote of appreciation was given, of this method of instructing the people and children, and its adoption by others of the profession recommended.

Dr. Brown read an essay on Alveolar Abscess, a production replete with valuable information. A copy is in the hands of the committee on Publication.

ALVEOLAR ABSCESS.

Dr. Stone, treats by two methods, internal and external. First step is depletion, for relief of patient. Then open largely to apex of fang, if possible, introducing creosote to destroy the sack. If the creosote touches all points there is no fear of failure.

Dr. McClelland. First open to apex, if muscles of face are sore and swollen apply leaches to the gums which if applied in time will generally prevent the formation of pus.

If pus has formed and fistulous opening found, treat fangs and fill them, and treat from external opening by injecting iodine.

UTILITY OF TEMPORARY ARTIFICIAL TEETH.

Drs. Stone, Driggs, McClellan and others advocate their insertion.

Dr. Redman, opposed it on account of the tendency to thicken the alveolar ridge, thereby producing prominence of the lips.

Dr. McClelland thinks that the absence of teeth causes the lips to become unnaturally thick and rigid.

